# Humanitarian action in academic institutions: a case study in the ethical stewardship of unidentified forensic cases

**DOI:** 10.1080/20961790.2022.2035063

**Published:** 2022-03-14

**Authors:** Justin Z. Goldstein, Mariah E. Moe, Emilie L. Wiedenmeyer, Petra M. Banks, Sophia R. Mavroudas, Michelle D. Hamilton

**Affiliations:** Department of Anthropology, Texas State University, San Marcos, TX, USA

**Keywords:** Forensic sciences, forensic anthropology, unidentified persons, long-term unidentified, identification, academic institutions, standardisation, humanitarian action

## Abstract

Forensic anthropologists are often responsible for the management of long-term unidentified individuals. Others have contextualised these decedents—many of whom likely belonged to socially, politically, and/or economically marginalised groups in life—as part of a larger identification crisis in the US. However, there has been little discussion surrounding how this humanitarian crisis has manifested in academic institutions, where anthropologists often provide medicolegal consultation and act as long-term stewards of the unidentified. The Identification & Repatriation Initiative was created at the Forensic Anthropology Centre at Texas State University (FACTS) to recognise and investigate unidentified human remains in long-term storage. Our paper outlines common challenges that were encountered during our initial reassessment of unidentified cases at FACTS, emphasising the detrimental impacts of inconsistent procedures, loss of context, and case fatigue. It is likely that other academic institutions face similar challenges, and by highlighting these issues we hope to help initiate a larger conversation concerning ethical stewardship of human remains in these settings. By incorporating humanitarian perspectives into forensic casework, anthropologists in academia can better advocate for the long-term unidentified.

**Key points**
- Forensic anthropologists at academic institutions are qualified to act as consultants on forensic casework when requested by jurisdictional authorities and are often responsible for the long-term management of unidentified human remains.
- The long-term unidentified represent a vulnerable population and academic institutions are not exempt from calls for humanitarian approaches to identification.
- The Identification and Repatriation Initiative was created at the Forensic Anthropology Centre at Texas State University to acknowledge and investigate unidentified human remains in long-term storage.
- This paper considers possible ways for humanitarian action to be incorporated into academic settings and suggests anthropologists can better advocate for the unidentified through procedural standardisation, institutional and interagency collaboration and ethical stewardship.

## Introduction

The professional responsibilities of forensic anthropologists frequently include working with unidentified human remains. Indeed, the history and development of forensic anthropology, particularly in the US, has been intricately linked to government sponsored identification efforts [1], such as those now spearheaded by the Defense POW/MIA Accounting Agency (DPAA) [2]. In some cases, unidentified remains are identified relatively quickly by filtering missing persons lists using standard biological profile results or by utilising advances in DNA processing [3], comparative medical or dental radiography [4], and other traditional analyses. In other cases, particularly when the unidentified remains belong to socially, politically, or economically marginalised populations, the identification process may take much longer. Marginalisation, defined here as a process of peripheralisation that can include an individual’s loss of identity or separation from context [5], may result from political, social, economic, and environmental factors. Individuals that frequently remain unidentified in death due to these influences can be seen as continuing to embody the marginalisation that they experienced in life. An example of this differential treatment of the marginalised dead has been clearly observed by those working along the US–Mexico border to identify and repatriate the remains of presumed migrants who perished along the border, where the influences of structural violence are omnipresent [6–11]. Because such decedents often had limited resources in life, they frequently do not have robust antemortem medical records that can aid in the identification process [12–13]. Thus, these unidentified individuals and their communities are repeatedly subjected to oppressive systems of inequity and vulnerability that cause suffering, reify inherent structural inequalities, expand risk, and limit opportunity along social axes, even in death [10].

That the identification process is inherently political is in no way a novel concept. Because the success of identification efforts relies largely on the allocation of economic, social, and individual resources, it is impossible for such work to completely disengage itself from political biases. Such a shift in the role of the forensic anthropologist is centered around humanitarian forensic action, defined by Cordner and Tidball-Binz [14] as the application of forensic medicine and science in humanitarian contexts. Recent work [15–17] has highlighted the need for forensic anthropologists working with medicolegal agencies across the US to expand their roles and adopt an explicitly biocultural, humanitarian approach to forensic casework. Goad [17] calls for forensic anthropologists to recognise not only the increasing numbers of long-term unidentified cases as a humanitarian crisis, but also to recognise an obligation to document and disseminate inequalities in the identification process. Thus, although humanitarian action is pursued neutrally, its application in the identification process is able to illuminate politically driven inequalities. Given that unidentified human remains from undomiciled, transient, and other vulnerable populations make up a significant portion of casework for medical examiner and coroner offices across the US [18–20], it is important to consider how such decedents, even in isolation, are connected by larger systems of structural violence and vulnerability. Deeper engagement is required regarding identification as a human right that persists in death, and how lived experiences of inequality are embodied in long-term unidentified remains [10,17].

This is especially important for forensic anthropologists working in academic settings, who often act as external consultants for medicolegal agencies and jurisdictional authorities. In these circumstances, anthropologists are not only responsible for the initial analysis of skeletal remains, but also are frequently asked to accept custody of these remains for long-term storage. Rather than accepting cases for long-term storage without hesitation, academic institutions should consider whether accepting cases is ethical based on the availability of physical resources (e.g. laboratory and/or curation space, operating budgets) and time investments that practitioners in academia can feasibly commit towards the analysis, identification, and active stewardship of remains. In scenarios where this is not feasible, exploring collaborative options with other institutions may be beneficial to the analyst and identification of the unidentified individual alike. In short, accepting cases for long-term storage at academic institutions comprises not only a promise to take responsibility for the physical remains, but also a commitment to take responsibility for pursuing a positive identification regardless of how long that process may take. Especially in cases where identified individuals who remain unclaimed are brought to academic institutions, long-term storage requests should be denied and medicolegal agencies should determine final disposition. Similarly, remains that are not forensically significant should be referred to local jurisdictional authorities that oversee historic remains. However, for unidentified forensic cases, long-term storage and re-analysis is arguably a preferable alternative to cremation or inconsistently documented burials, both of which may severely limit the potential avenues for a positive identification. Unfortunately, such practices are common throughout the decentralised medicolegal systems across the US, including the jurisdictions with which Forensic Anthropology Centre at Texas State (FACTS) interacts.

### The formation of the FACTS identification and repatriation initiative

The FACTS was formally established in 2008 to develop an academic programme within the Department of Anthropology, Texas State University focused on training students in forensic anthropology and centered around an outdoor human decomposition facility and associated willed body donation programme. Prior to the development of this focused forensic anthropology programme, individual faculty members and their students would perform casework and consult with local jurisdictions as requests for assistance were made, maintaining individual records of case reports with no standardisation of data collection, analysis methods, report formatting, or evidence maintenance. Fortunately, since the incorporation of FACTS in 2008, faculty and staff have maintained casework files, reports, and accessioned remains (despite inheriting an inconsistent case numbering system). In 2011, the university formally appointed a permanent Director of FACTS. Today, FACTS is often responsible for the management and housing of forensic anthropology cases, accepted with explicit approval from jurisdictional authorities (see Supplementary material for an example FACTS authorisation form). These individuals are not accessioned into the Texas State Donated Skeletal Collection as teaching resources or for research, but are kept in a separate, secured room where they are meant to be stored until a positive identification is made. Ideally, FACTS acts as a temporary steward for all these remains, facilitating an identification and then returning the individual to the custody of family representatives or the proper jurisdictional authority. However, for cases that do not have a quick resolution, this temporary housing can transition into long-term storage. As such, they become a part of the larger humanitarian crisis described by Goad [17], meriting a more engaged approach. It is vital that an ethos of ethical stewardship is developed for such settings, wherein the decedent is prioritised.

For the cases since 2008 at least, there are records of which counties consulted with FACTS for casework and some files associated with the anthropological reports generated by FACTS faculty. However, forensic anthropology casework had been done at the university for at least 30 years before the establishment of the centre and, while some of the physical evidence for those cases remain at FACTS, for some of these cases there exists no written record of which counties, states, or jurisdictions those cases came from. Furthermore, for some there are no records of analysis of the remains or formal reports. Since the current director was put in place in 2011, several punctuated efforts have been made by the director and faculty to catalogue these case files and associated skeletal material, including attempts to recover the documentation associated with some of the pre-2008 cases.

To this end, the FACTS Identification & Repatriation Initiative (FACTS IRI) was established in 2020 by approval of the FACTS Board to reexamine all long-term unidentified cases currently managed by FACTS, which date back to the 1960s. We identified three common challenges that have hindered identification efforts for these individuals, which we have categorised as 1) inconsistent procedures, 2) loss of context, and 3) case fatigue. It should be noted that the challenges described in this manuscript are not universal to all casework occurring in forensic or academic institutions, as each facility is unique in its resources and personnel, as well as its strategies and decisions. That said, by documenting and disseminating these challenges, we hope to promote a collective discussion about finding potential solutions to common identification issues and explore how a humanitarian approach can be integrated into forensic casework at academic institutions. Improving standardisation, fostering institutional and interagency collaboration, and committing to ethical stewardship can help forensic anthropologists better advocate for the long-term unidentified. In addition, we also hope that our experiences can provide other institutions with an example of how these cases might be addressed.

## Inconsistent procedures

Standardised documentation and procedures are fundamental to maintaining proper upkeep and oversight of daily forensic casework. During our reassessment, we encountered significant inconsistencies in case file documentation procedures, especially with cases accepted prior to the formal establishment of FACTS. This included inconsistent case numbering, missing recovery reports and case information, as well as missing or incomplete data collection sheets. Crucially, this also included inconsistent documentation of DNA collection, DNA submission, entries into the National Missing and Unidentified Persons System (NamUs—an online resource for US-based investigative agencies and families of the missing to post cases and compare potential identifications), and case-related communications. These issues not only make attempts to revisit previous casework overwhelming, but they also mean that such attempts will require significant time investments and unnecessarily duplicative work. The reality is that for many institutions, such efforts may be impractical, particularly given the lack of dedicated personnel and the demands of newer cases.

At FACTS, past attempts to reengage with the long-term unidentified cases and initiate standardised procedures stagnated, in part, because of the issues described above. Additionally, with personnel turnover came disruptions to the implementation and consistent use of these protocols. Prior attempts included changes to the case numbering system, the addition of a workflow checklist with standardised basic data collection sheets, and an attempt to standardise NamUs entries. These initiatives were undoubtedly beneficial; however, they were not maintained consistently among analysts. Over time, this resulted in inconsistent and poorly documented case numbers, which made reassessing cases logistically challenging and time consuming. Moreover, it was unclear what steps had been completed in prior investigations. For example, although NamUs entry forms were printed for most cases, they were rarely completed, and NamUs Unidentified Persons (UP) numbers were often not documented. While these workflow checklists marked an important step in the right direction for long-term unidentified case management, they were not successfully incorporated into standardised procedures for analysis, ultimately making it difficult for subsequent analysts to access basic case information. Put briefly, these issues can be summarised as a failure to standardise workflow and personnel in case management.

General protocols established through standard operating procedures (SOPs) can streamline decisions on current institution-preferred and field-approved forensic methods, while allowing for flexibility in case analysis by the practitioner. SOPs can and should detail what data to record and permanently store at the facility, as it not only influences the quality of contextual information available for each case, but also dictates which cases can be properly reassessed over time. A lack of such contextual information can create intimidating hurdles when later assessing what investigative avenues remain for the long-term unidentified. Establishing standardised protocols can be particularly challenging in newly established institutions; however, regardless of casework volume, initiating the process towards standardisation prevents confusion and mistakes down the line. Forensic casework can quickly become overwhelming and having standard procedures in place allows for controlled and well-managed case analysis during periods of high caseload.

Maintaining detailed SOPs in the lab is vital to preserving the consistency of forensic work and all efforts should be made to ensure that the quality of collecting, recording, and submitting forensic data is kept at the highest level possible. This is especially true for cases in long-term storage. A review of such cases should follow institution-based procedural standards while maintaining documentation throughout the process. This can be accomplished through the implementation of a workflow checklist kept with the casefile in which each stage of the investigation is documented in a standard manner, tracking the case number, analyst, date of collection, and any pertinent notes. Standard items for documentation should include time stamps of when skeletal data collection and biological profile estimations were completed, information regarding photographs of the case including any possible identifying markers, DNA collection, DNA submission, NamUs (or comparable database) entry and records of associated NamUs case numbers, along with the results of any supplementary investigations. Although each institution will differ in what types of data are collected, all institutions should have minimum requirements for case documentation to avoid problems of personnel turnover. An effective system is one that requires appropriate training, qualifications, and/or field experience to maintain rigorous case documentation that can be logically understood by all involved regardless of timeframe or personnel changes.

## Loss of context

The most striking complication encountered during our reexamination of the FACTS long-term unidentified cases was the overwhelming loss of case context that had occurred throughout the decades. These losses included principal case information (e.g. provenance, county, year of discovery), institutional knowledge about decision-making on behalf of the case, first-hand knowledge of the recovery sites, and even the names of original analysts (e.g. initials were present, but the original analyst could not be identified from those initials). Knowing what information to record and in how much detail is critical as forensic recovery scenes are permanently altered once an investigation begins. Excavation, maceration, and bone sampling are irreversible and destructive activities, making careful documentation important for avoiding complications in all future analyses. The quality of contextual information remaining (either through loss of context over time or a lack of initial contextual data collection) is dependent on the experience of the investigators, law enforcement, and anthropologists alike. While loss of contextual information can occur because personnel failed to collect or document that information initially, another common situation we observed was the gradual loss of context as data are transferred between personnel over time. Institutions often go through personnel changes, particularly over long periods of time, but every iteration has the potential to further erode contextual information if not properly documented and maintained. Long-term cases appear particularly vulnerable to case manager changes as analysts relocate, retire, or are reassigned.

At FACTS, the most salient example of this challenge came from casefiles with no available contextual data. It was determined that, while contextual data were once available, all original documentation and analyses were removed from FACTS during personnel turnover. Moreover, all these cases had been labeled with case numbers that reflected an analyst’s initials, rather than with a standardised accession case number that could be used to determine the year, the county, or even the jurisdictional authority associated with the recovery. Thus, the only remaining information available was what could be analysed from the isolated skeletal remains.

In academic institutions, it is natural that faculty members consulting on forensic casework will eventually retire and students assisting with casework will graduate and move on. As such, these types of changes should be expected and anticipated. Any transition should involve a thorough transfer of active cases and casework to incoming parties, including case reports, data sheets, and any pertinent notes that could assist in identification. It is important to emphasise that the transfer of data and documentation to new personnel should be centered around collaboration towards an identification. When data concerning the recovery context go missing during personnel changes, new analysts are denied the opportunity to review this associated contextual data, which significantly impacts the decedent’s potential to be identified. One of the primary goals of a forensic anthropologist, both ethically and professionally, is the identification of the unidentified and the prioritisation of the decedent in the identification process. Increasing the possibilities for identification should be placed before any opportunities for professional growth (e.g. increasing an individual’s case experience) and unidentified forensic cases should never be confused with institutional resources (i.e. teaching collections). Care should also be taken to best preserve unidentified remains during analysis and storage, as any decision to permanently alter the remains (e.g. label the skeleton using permanent ink) may be seen by loved ones when the individuals are successfully identified and repatriated [21]. Any views of “ownership” over cases, case reports, or contextual data reduce forensic cases to mere objects, which ignores all social, emotional, and political connections that human remains still have in society after death. The separation between individuals and their identity also severs the link between individual and community, enacting a form of social death that prevents social healing and mourning that would typically occur by physically or ritually interacting with an individual’s memory [22]. The ultimate harm is thus multi-fold: withholding crucial case information and not prioritising the decedent disrespects the unidentified, their next-of-kin, and their communities while the identification process is halted or unnecessarily delayed.

When approaching cases that have missing contextual data, we have found that interagency collaboration can be a powerful solution. Local jurisdictional authorities should have external records of consultations as well as their own case reports, meaning that case context can be reobtained from their sources. Even in scenarios where original case numbers are not available, medicolegal agencies may be able to find potential casefile matches if provided with basic biological profile demographics. For this reason, older FACTS cases are being reanalysed using more recently validated forensic methods (e.g. updated statistical models for biological profile analysis, forensic genealogy, and stable isotope analysis, all dependent on available resources). We will also be adopting this approach at the suggestion of and in collaboration with the Texas Rangers (a state-level law enforcement agency in Texas) who can disseminate “Be On the Look Out” (BOLO) notifications to surrounding jurisdictions and all 254 Texas counties. Although more commonly utilised in wanted suspects and dangerous persons alerts, these particular BOLO notifications will include all available case numbers, approximate timeframe received, decedent demographics, DNA submission status, NamUs case status, and any photographs that may help reconcile the remains in our custody to the original reporting law enforcement agency or jurisdictional authority. Despite a lack of available contextual information, adopting new identification strategies from beyond anthropology can help reinvigorate seemingly unapproachable long-term unidentified cases.

## Case fatigue

Stagnation and case fatigue represent dangerous yet common challenges when faced with long-term forensic cases. Given the significant time and resource investments needed to facilitate an identification, it can often be years before repatriation is possible. If traditional forensic anthropological methods fail to provide an identification, an unclear path forward can easily suspend further work on the case. Once all possible efforts have been exercised in investigating a case, it can become discouraging to continue investing time and resources into cases that appear to have a low chance of resolution. This is made more challenging when faced with limited resources and an accumulating caseload. Despite these issues, all open forensic cases should be afforded equal time, resources, and efforts upon acceptance as forensic casework. In addition, as forensic cases are transitioned into long-term storage, they should continue to be investigated at regular intervals as they often represent some of the most vulnerable populations in life as well as in death [17]. Standardising the practice of re-analysis may not only benefit identification efforts, but also reframe how analysts treat (subconsciously or otherwise) long-term unidentified cases. It is our position that if a forensic anthropologist chooses to accept unidentified cases, this act should acknowledge the potential for long-term storage, during which they are responsible not only for identifying these individuals, but also for actively advocating on their behalf.

For forensic anthropologists working in academic institutions that do not serve as the jurisdictional authority for a case, it can be easy to simply submit the initial report and consider the job complete without following up on any potential developments. If the case is stored with the forensic institution pending identification, this follow-through is necessary to avoid storing cases in perpetuity. New developments—such as a new investigator, innovative technology, or advancement in forensic methods—could help reinvigorate efforts toward identification and case resolution. Additionally, it is important to emphasise that the language used when describing and discussing long-term forensic cases can be a powerful tool in influencing the mindset and drive of forensic investigators [16]. Careful language should be used in both written documents as well as in verbal discussions to promote positive thinking, while maintaining realistic and achievable goals for identifying each individual. This includes avoiding terminology such as “cold” case, which is ultimately harmful to the victim, the family, and communities still invested the individual’s eventual return home, as it suggests a lack of concern and attention [23]. Specific protocols should be put in place to mandate what other strategies and steps should be taken to keep actively pursuing case resolution.

The FACTS IRI was developed to exercise this follow-through by reengaging and reassessing our long-term unidentified cases. During the initial phase of reassessment, we found several instances where lines of communication between anthropological personnel and the investigating agency faded and eventually stopped, especially if any personnel involved with these cases had left either the institution or the original investigating agency. Many cases also appear to have been entered into NamUs and/or had DNA samples collected and submitted to the University of North Texas Center for Human Identification for genomic profiling, but there is a lack of clear information about these efforts in the casefiles. It is particularly tempting to rely on undocumented institutional memory for case information, but readily accessible physical and digital documentation should always accompany the wealth of information that current personnel can provide.

Many of the issues that accompany the lack of follow-through are intrinsically tied to a lack of knowledge of the current state of the investigation and the resources available for case follow-up. Effective case management relies on standardised documentation that records all the necessary steps in the DNA submission process, NamUs entry, and anthropological analysis. Perhaps most importantly, there needs to be routine correspondence with the investigating agency, not only to provide them with any anthropological case updates, but also to receive new developments that may aid in the identification of the decedent. Of course, not all anthropological analyses should be identical—each analyst should be able to determine the methods required to best assess the case at hand. However, by standardising and communicating the basic components of case management, the institution assumes responsibility for managing case fatigue rather than the individual analyst. Moreover, this approach assures institutional consistency while allowing for individual flexibility in analyses.

To maintain consistency from here into the future, FACTS has instituted competency examinations for all students who want to be involved in casework, including analysis of the long-term unidentified. Through the establishment of clear competency guidelines (Supplementary material), FACTS is able to ensure consistency between generations of graduate students and simultaneously take advantage of the annual replenishment of new students who bring renewed enthusiasm and fresh eyes to long-term unidentified cases.

## Conclusions and future recommendations

Moving forward, it is vital that an ethos of stewardship is developed for forensic anthropologists who analyse and manage unidentified remains, including those investigating and managing cases in academic contexts. While some of the barriers to identification discussed in this paper may be unique to our institutional history, it is likely that other academic programmes with their own open forensic casework will recognise similar complications that may impede identification of the long-term unidentified. By highlighting the challenges we have encountered, we hope to catalyse conversations concerning ethical stewardship of human remains in academic settings. Approaching these decedents from a humanitarian perspective underscores the need for case managers and analysts to integrate theoretical, ethical, and technical considerations into their practice. We have begun this reassessment to examine past custodial practices that treated skeletal remains contrary to our current ethical standards, to identify procedures that have hindered the identification process, and to formally standardise analytical and documentation protocols. This work—informed by calls for a humanitarian approach to identification of the long-term unidentified—has helped develop a more holistic engagement that aims to meaningfully advance identification initiatives while maintaining obligations to stakeholders and respecting the agency and dignity of unidentified individuals.

In addition, when reassessing the long-term unidentified individuals, we encountered remains of individuals from archaeological Native American contexts. Although archaeological remains and forensic cases are typically addressed in separate jurisdictional settings within the US, the two contexts commonly coexist in academic institutions as law enforcement may bring in recovered skeletal remains for assessment of medicolegal significance (i.e. archaeological, historic, or forensic). It is the position of the FACTS IRI that there is an ethical obligation to explore repatriation and final disposition (e.g. burial), even for unprovenanced archaeological Native American remains with unknown or unclear group affiliations. We intend to explore options for the repatriation of these individuals to descendent communities (if known) or to tribal representatives as we work to reassess the forensic cases. Although it is outside the scope of this paper to discuss all the challenges that ethical stewardship of archaeological remains entails, it is important to acknowledge the limits of forensic expertise. A crucial component of ethical engagement with human remains is understanding the legal and humanitarian obligations to descendent communities, including when to consult additional experts. This engagement can be incorporated into academic institutional policies by reconsidering what types of remains are appropriate to accept for analysis and retention, or by initiating partnerships with tribal and archaeological experts who can consult on repatriation initiatives. Future conversations should explore how archaeological remains, particularly in academic forensic settings, are vulnerable to comparable systems of marginalisation as those seen in long-term unidentified forensic cases.

In light of our experiences, we offer the following suggestions for consideration by academic institutions that manage unidentified human remains and those who may be confronting similar issues:

1. Institute SOPs that include the following recommendations for case analysis and long-term management at the institutional level, as well as continuing to review and renew such documents to address any necessary updates. FACTS has formalised standards by adapting publicly available operating procedures and quality assurance protocols codified by the New York City Office of Chief Medical Examiner Forensic Anthropology Unit [24], as well as best practices published by the Scientific Working Group for Forensic Anthropology (SWGANTH) [25].
2. Document and preserve contextual information.
3. Anticipate and facilitate complete transfer of case documentation between analysts over time as necessary.
4. Incorporate routine case reassessments into standard workflow protocols, applying newly validated analytical methods with the support of certified student populations. Competency guidelines and certification requirements employed by FACTS are provided as Supplementary material.
5. Initiate cooperative partnerships with local jurisdictional authorities who are familiar with their communities and can bring a new perspective or approach to casework.
6. Collaborate with other local academic institutions that manage unidentified human remains to continually improve SOPs and workflow protocols.
7. Consider and codify what types of remains are appropriate to accept, developing relationships with appropriate expert consultants.
8. Build relationships with familial and community stakeholders who are invested in the solvability of missing persons cases and the identification of unknown remains.

Although these items appear straightforward, they are not often codified into academic practice, and if put into standard protocols, they could each work towards expanding communication between anthropologists in academic settings and the medicolegal systems in which they are embedded. This is especially pertinent in the US where forensic anthropologists practicing in academic institutions are important contributors to the varied medicolegal systems in place. Emphasising the humanitarian obligation to not only engage with existing ethical and logistical difficulties, but also to anticipate future challenges is a critical step towards developing an ethos of ethical stewardship of the long-term unidentified.

## Supplementary Material

Supplemental Material
