# Supplementary material for: Humanitarian action in academic institutions: a case study in the ethical stewardship of unidentified forensic cases
Source: Forensic Sci Res. 2022 Mar 14;7(3):358–65. doi: 10.1080/20961790.2022.2035063 (PMC9639541; doi:10.1080/20961790.2022.2035063)
Supplement: Supplemental Material [file TFSR_A_2035063_SM7440.zip › SupplementaryMaterial_A.pdf]

TEXAS STATE®
FORENSIC ANTHROPOLOGY CENTER

# Justice of the Peace Authorization for Release of Unidentified Human Remains

I, Justice of the Peace, ______________________________, representing __________________
print name
County, Texas, Precinct # _______ authorize the transfer of unidentified human remains representing agency case number ______________________ to the Forensic Anthropology Center at Texas State (FACTS) for forensic anthropological analysis and identification efforts.

☐ By checking this box, I (the Justice of the Peace) request that FACTS curate the above-mentioned case pending identification.

Justice of the Peace: ______________________________ Date: __________________
signature

Remains released to: ______________________________ Date: __________________
print name

______________________________
signature

FACTS Case No.: ______________________________

Additional Notes:
