# Supplementary material for: Humanitarian action in academic institutions: a case study in the ethical stewardship of unidentified forensic cases
Source: Forensic Sci Res. 2022 Mar 14;7(3):358–65. doi: 10.1080/20961790.2022.2035063 (PMC9639541; doi:10.1080/20961790.2022.2035063)
Supplement: Supplemental Material [file TFSR_A_2035063_SM7440.zip › SupplementaryMaterial_B.pdf]

FACTS Field and Casework Teams

Faculty/Program Faculty

FACTS Faculty/Program Faculty are classified as either FAII or FAIII according to FACTS guidelines (see Appendix I: FACTS Qualifications – Minimum Standards). Faculty/Program Faculty author case reports, lead search and recovery teams, create and review exams, and designate member assignments. All Faculty/Program Faculty should be notified of a forensic case via email or phone in a timely manner.

Peer Review: Each case or request for assistance will be supervised by assigned FACTS Faculty/Program Faculty, who will be responsible for ensuring the case is completed, peer reviewed, and submitted to the appropriate authorities in a timely manner. All case reports must be peer reviewed by an FAIII prior to final submission.

Students

- Doctoral students may achieve either Assistant or Full Member status, depending on exam results and experience.
- Master’s students may achieve either Trainee or Assistant Member status, depending on exam results and experience. Master’s students cannot participate as CAT Assistant Members until they have passed Skeletal Methods I and Skeletal Methods II *with a grade of A in each class*. Please note, however, that not earning an A in both Methods classes *does not* prohibit participation in casework as a Trainee Member of the CAT; it simply limits authorship.
- Undergraduate students who are FACTS-affiliated volunteers or interns may achieve Trainee Member status, depending on exam results, experience, and approval of Team Leaders.
- Operation Identification Participation: All FACTS Forensic Team qualifications apply

FACTS
Search and Recovery Team

There are two categories of student participation possible in FACTS forensic casework:

1. Search and Recovery
2. Case Analysis

1. Search and Recovery Team (SRT)

SRT Trainee Members

- *Requirements*: Must be FACTS-affiliated graduate students or FACTS student volunteers/interns. Must pass the “Basic Human Osteology” exam.
- *Abilities*: Participate in FACTS Search and Recovery efforts under the supervision of an Assistant or Full member (see below)

SRT Assistant members

- *Requirements*: Must be FACTS-affiliated graduate students. At a minimum, must be at least an FA I (see Appendix I). Must pass “Basic Human Osteology,” “Advanced Human Osteology,” and “Map Making” exams.
- *Abilities*: Participate in FACTS Search and Recovery efforts, direct trainee members in the absence of a Full member, map scenes, and carry out additional responsibilities as assigned by a Full member

SRT Full Members

- *Requirements*: Must be at least an FA II (see Appendix I). Must pass “Basic Human Osteology,” “Advanced Human Osteology,” and “Map Making” exams. Must have completed or taught a human remains excavation course that included surface scatter and burial recovery.
- *Abilities*: Lead FACTS Search and Recovery efforts in the command position; can excavate, record and recover human remains, direct Trainee and Assistant members

Notes:

1. Owing to minimum qualification standards as defined by FACTS guidelines (see Appendix 1), MA-seeking students can never become Full members.

2. Both Assistant and Trainee memberships can be entered into directly if the requirements are met.

3. For Search and Recovery efforts, all students will be on a rotation schedule, and the only acceptable excuse for skipping your turn is if you have class during that time or you have a university-approved excuse. Otherwise, if you miss your rotation you are moved to the back of the rotation.

2. Case Analysis Team (CAT)

CAT Trainee Members

- *Requirements*: Must be FACTS-affiliated graduate students or FACTS student volunteers/interns. Must pass the "Basic Human Osteology" and "Skeletal Inventory" exam.
- *Abilities*: Lay out skeletal remains for forensic case analysis; take photos

CAT Assistant Members

- *Requirements*: Must be FACTS-affiliated graduate students. Must be at least an FA I (see Appendix 1). Must correctly complete a biological profile of an individual from the Texas State Donated Skeletal Collection. Must pass the "Basic Human Osteology" and "Advanced Human Osteology" exams.
- *Abilities*: Lay out skeletal remains for forensic case analysis and take photos. Co-author biological profile case sections under the direction of a Full member, or co-author a full case report under the direction of a Full member

CAT Full Members

- *Requirements*: Must be at least an FA II (see Appendix 1). Must have completed two co-authored/submitted case reports with a FACTS Faculty member while an Assistant Member. Must pass the "Basic Human Osteology" and "Advanced Human Osteology" exams.
- *Abilities*: Single author case reports for submission, or direct Assistant members in co-authoring submitted case reports. All reports must be peer-reviewed by a Team Leader.

Notes:

1. Owing to minimum qualification standards as defined by FACTS guidelines (see Appendix 1), MA-seeking students can never become Full members.

2. Both Assistant and Trainee memberships can be entered into directly if the requirements are met.

3. Full membership in the Case Analysis Team cannot be entered into directly by PhD-seeking candidates. They must first progress through the Assistant membership category, where they will complete two co-authored case reports with a FACTS Faculty Member before they can move up to Full Members.

Operation Identification

Operation Identification Activities

- *Requirements*: Must meet appropriate FACTS SRT and CAT member levels for participation.
- Non-FACTS Team members can participate as interns/volunteers at the discretion of the OpID director
- Processing for OpID is open to volunteers
- Some OpID activities may require specialized OpID exams and/or training activities

.

FACTS Forensic Team
Exams

Testing will be offered at least once a year at the end of the Fall Semester, and by appointment at limited times throughout the rest of the year. FACTS Team Leaders will schedule and administer testing. An individual may retake a failed exam up to two more times. Failure to pass an exam during all three attempts will result in permanent exclusion from all failed-level activities.

Basic Human Osteology Exam

- Identify whole elements including side (left vs right), distinguish between adult/subadult skeletal material
- 20 stations
- Passing is 16/20 (80%) or higher

Advanced Human Osteology Exam

- Identify fragments as to element and side
- Can include nonhuman elements (nonhuman elements correct answer = nonhuman)
- 20 stations
- Passing is 16/20 (80%) or higher

Map Making Exam

- Correctly use baseline mapping techniques to map in an assigned surface scatter of 3 elements and produce an accurate map. Correctly trilaterate from the baseline to 2 predetermined datum points.
- Accurate maps will include at least one point for each element, a title, a north arrow, a scale, a key, the date, and the name of the person who made the map.
- Passing is correct mapping of all 3 elements along with a correct title, north arrow, scale, key, and correctly describing the position of each datum in relation to the baseline.
- Suggested Reading: Christensen, A. M., Passalacqua, N. V., & Bartelink, E. J. (2019). Chapter 6 - Forensic archaeology and scene processing methods. In A. M. Christensen, N. V. Passalacqua, & E. J. Bartelink (Eds.), *Forensic Anthropology (Second Edition)* (pp. 183–215). Academic Press. https://doi.org/10.1016/B978-0-12-815734-3.00006-3
-

Skeletal Inventory Exam

- Correctly lay out a skeleton from the Texas State Donated Skeletal Collection (TXSTDSC) in anatomical position and complete a Skeletal Inventory Exam Sheet.
- The skeleton must include at least four teeth, hands, and feet.
- Accurate inventories will correctly identify the teeth as to type (I, C, P, or M), whether it is upper or lower, and the side. Accurate inventories will correctly side all elements (except phalanges) and record an accurate number of phalange type.
- TXSTDSC donations should not be used if they are highly fragmented or exhibit excessive trauma/pathology.
- Reference books are permitted for use during the Skeletal Inventory Exam.
- A FACTS Team Leader must sign off on the completed exam.

Biological Profile Requirement

FACTS Forensic Team
Exams

- Can be complete during coursework (Methods II or PhD Methods)
- Requires a TEAM Leader to sign a Biological Profile Requirement Form

Note: Any FACTS Team Leader, in consultation with other FACTS Team Leaders, may set up additional proficiency or competency specialized exams to administer testing in specific areas (i.e., histology, digitizing, radiography, etc.).

APPENDIX 1:
FACTS "Qualifications – Minimum Standards" Guidelines (adapted from SWGANTH)

The SWGANTH "Qualifications – Minimum Standards" (2014) draft document outlines qualification standards for practicing and professional forensic anthropologists. NIST OSAC is currently using this document to draft minimum qualification standards; therefore, until a final document is approved, this draft document will be the guiding framework used by FACTS to assign student and practitioner levels based on minimum levels of education, training, experience, and professional activities required to practice forensic anthropology at various levels across the United States. Please note: some of the qualifications have been edited to fit FACTS requirements.

1.0 Principle, Spirit and Intent
Establishing qualifications to practice forensic anthropology ensures that the best interests of the profession, the justice system, and society are served. Practitioners shall possess the appropriate qualifications relevant to the forensic anthropology-related duties they are expected to perform.

2.0 Purpose and Scope
The following guidelines indicate minimum levels of education, training, experience, and professional activities required to practice forensic anthropology at various levels. These guidelines apply to forensic anthropologists practicing in any laboratory setting including medicolegal facilities, universities, law enforcement agencies, government laboratories, and other crime laboratories.

Laboratories shall employ the most qualified forensic anthropologists appropriate to its case-working functions and organizational requirements. These guidelines are based largely on US education and training systems; outside of the US, consideration should be given to the availability of comparable education and professional development programs. In the absence of specific guidelines or procedures, the principle, spirit and intent should be met.

3.0 Forensic Anthropologist I (FA I)
A Forensic Anthropologist I works under close supervision of a Forensic Anthropologist II or III. Responsibilities may include data collection (e.g., taking measurements and notes), assisting with forensic anthropological analyses, and co-signing reports. A Forensic Anthropologist I shall possess the following qualifications:

- A Master's Degree (MA or MS) or equivalent in Physical/Biological Anthropologyor closely-related field, with a significant emphasis on skeletal biology, human variation, human anatomy, and statistics;
  OR
- A Bachelor's Degree (BA or BS) or equivalent, and active enrollment in a graduate program with a Forensic Anthropologist III, with similar emphasis

- Participation in relevant continuing education and training (e.g., workshops, short courses) in forensic anthropology or related fields. Presenting research at professional meetings such as the American Academy of Forensic Sciences.

4.0 Forensic Anthropologist II (FA II)
A Forensic Anthropologist II independently conducts forensic anthropological analyses and writes and signs reports. A Forensic Anthropologist II shall possess the following qualifications:

- A Master's Degree (MA or MS) or equivalent in Physical/Biological Anthropology or closely-related field, with a significant emphasis on, human osteology, skeletal biology, human variation, human anatomy, and statistics

- Active certification in forensic anthropology by a Forensic Specialties Accreditation Board-accredited organization (if and when available)

- Participation in relevant continuing education and training (e.g., workshops, short courses) in forensic anthropology or related fields

- Continuing professional development in forensic anthropology including one or more of the following:
    - Conducting research and authoring peer-reviewed publications
    - Mentoring Graduate Students
    - Teaching relevant courses at the collegiate level
    - Enrollment in a doctoral program

5.0 Forensic Anthropologist III (FAIII)
A Forensic Anthropologist III independently conducts forensic anthropological analyses and writes and signs reports. They may be the sole forensic anthropology practitioner in a laboratory, and may supervise individuals at the Forensic Anthropologist I or Forensic Anthropologist II levels.

A Forensic Anthropologist IIIA shall possess the following qualifications:

- A Doctoral degree (PhD) Physical/Biological Anthropology with a significant emphasis on forensic anthropology, human osteology, skeletal biology, human variation, human anatomy, and statistics

- Active certification by the American Board of Forensic Anthropology or other Forensic Specialties Accreditation Board-accredited organization (if and when available)

- Participation in relevant continuing education and training (e.g., workshops, short courses) in forensic anthropology or related fields

- Continuing professional development in forensic anthropology including one or more of the following:
    - Conducting research and authoring peer-reviewed publications
    - Mentoring graduate students, FAI, and FAII
    - Teaching relevant courses at the collegiate level

A Forensic Anthropologist IIIB shall possess the following qualifications:

- A Doctoral degree (PhD) Physical/Biological Anthropology with a significant emphasis on forensic anthropology, human osteology, skeletal biology, human variation, human anatomy, and statistics

- Participation in relevant continuing education and training (e.g., workshops, short courses) in forensic anthropology or related fields

- Continuing professional development in forensic anthropology including one or more of the following:
    - Conducting research and authoring peer-reviewed publications
    - Mentoring graduate students, FAI, and FAII
    - Teaching relevant courses at the collegiate level

FACTS Forensic Team Checklist

**Yes/No**	SRT Trainee Member
Yes/No	Passed the Basic Human Osteology Exam

**Yes/No**	SRT Assistant Member
Yes/No	Passed the Basic Human Osteology Exam
Yes/No	Passed the Advanced Human Osteology Exam
Yes/No	Passed the Map Making Exam
Yes/No	Completed a BA/BS

**Yes/No**	SRT Full Member
Yes/No	Passed the Basic Human Osteology Exam
Yes/No	Passed the Advanced Human Osteology Exam
Yes/No	Passed the Map Making Exam
Yes/No	Taught or attended a human remains recovery course that included surface and burial recovery
Yes/No	Completed an MA/MS

**Yes/No**	CAT Trainee Member
Yes/No	Passed the Basic Human Osteology Exam
Yes/No	Passed the Skeletal Inventory Exam

**Yes/No**	CAT Assistant Member
Yes/No	Passed the Basic Human Osteology Exam
Yes/No	Passed the Advanced Human Osteology Exam
Yes/No	Completed Bioprofile Exam
Yes/No	Completed a BA/BS
Yes/No	If a MA student, Completed Methods I and II with an ‘A’

**Yes/No**	CAT Full Member
Yes/No	Achieved CAT Assistant Status
Yes/no	Completed two co-authored Case Reports with a FACTS Team leader

Basic Human Osteology

- **20 Stations Total**
- **Passing is 16/20 (80%) or higher**
- **Identify whole elements including side, distinguish between adult/subadult**
- **Metacarpals/tarsals must be identified to ray # and side (e.g. left MC2)**
- **Teeth must identified as to type (I, C, P, M), upper or lower, and side. Nothing More.**
- **Vertebra must be identified to type (Cervical, Thoracic, Lumbar)**
    - **Cervical 1 (Atlas) and Cervical 2 (Axis) must be explicitly identified**

Photo Example	Element	Side
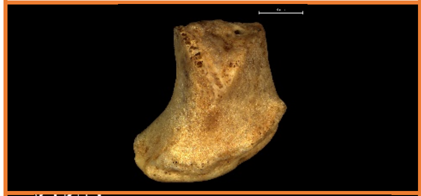	Trapezoid	Left
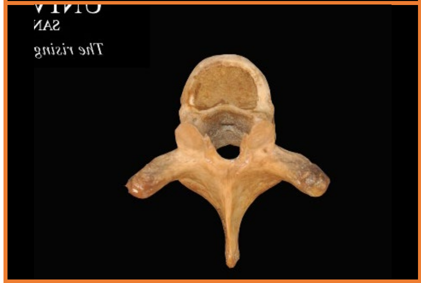	Thoracic Vert	midline
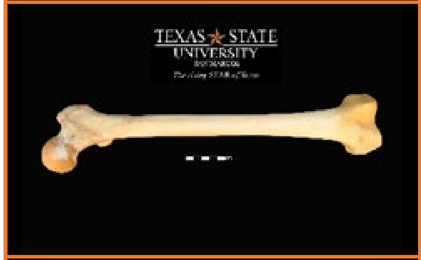	Femur	Left
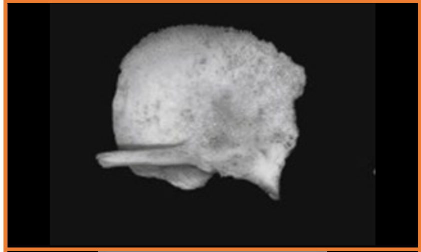	Subadult Temporal	Left
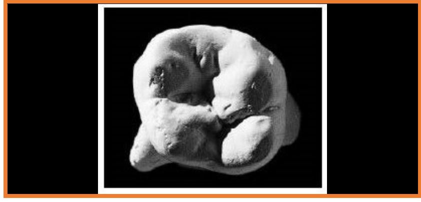	Upper Molar	Right

Advanced Human Osteology

- 20 Stations Total
- Passing is 16/20 (80%) or higher
- Identify fragments including side, distinguish between adult/subadult
- Metacarpals/tarsals must be identified to ray # and side (e.g. left MC2)
- Teeth must identified as to type (I, C, P, M), upper or lower, and side. Nothing More.
- Vertebra must be identified to type (Cervical, Thoracic, Lumbar)
    - Cervical 1 (Atlas) and Cervical 2 (Axis) must be explicitly identified
- Can include nonhuman elements (nonhuman elements correct answer = nonhuman)

Photo Example	Element	Side
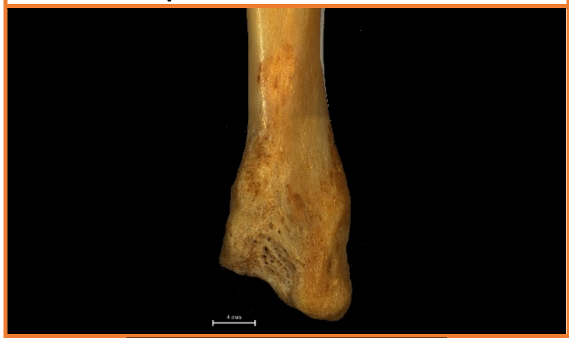	Proximal MC3	Left
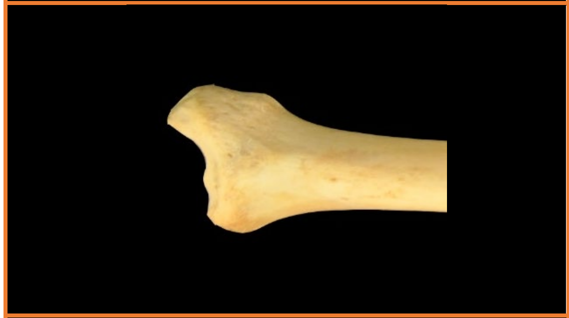	Distal Tibia	Left
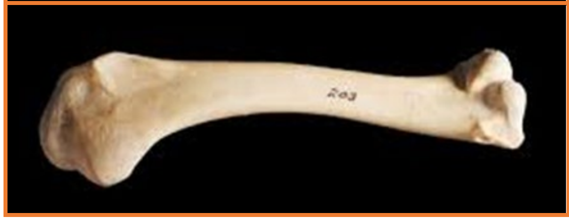	NONHUMAN	NONHUMAN

Mapping Test Form

Complete this form at the mock scene to ensure you have all the information you need to complete your map.

**Title of the Map**	**Surface Scatter of (case no.)________________________**
**Map Type**	**Baseline (with Trilateration of Datum)**
**Date**	
**Name of Recorder**	
**Description of the Datum**	**What is the datum (Reference point 1/RP1):**
	**If there is a subdatum (Reference point 2/RP2), description of the subdatum:**
	**If there is a subdatum, Distance between the datum and subdatum:** ____

**Use the Sections Below for the Map Measurements**				
**Description of the Baseline**	**Total Length of Baseline from Point A to Point B:** ____			
	**Direction: Baseline runs __________degrees E of North from point A**			
	**Directional Position of Baseline in Relation to Datum:**			
**Baseline Map Legend Information**	**Point**	**Description**	**X (cm)**	**Y(cm)**
	**1**			
	**2**			
	**3**			
	**4**			
	**5**			
**Trilateration of Datum Information**	**Distance between RP1 and RP2:**			
	**When using Trilateration RP1 is your datum and RP2 is your subdatum**			
	**Point**	**Description**	**Distance to RP1 (cm)**	**Distance to RP2 (cm)**
	**Baseline A**	**0,0 of baseline**		
	**Baseline B**			

Mapping Test Form

Draw an abstract overview of the scene below. Make sure to include a **north arrow**, **location of datum**, and **location of points** so you can confirm your map makes sense. On a real case you would have photos to refer to, but we are testing your ability to make a map based solely on your notes:

TEXAS STATE
Forensic Anthropology Center

Skeletal Inventory Exam
FACTS Forensic Team

Codes: 1 = present, complete   2 = present, fragmentary   3 = absent

**Cranium**	Left	Right		Left	Right
Frontal:	______		Maxilla:	______	______
Parietal:	______	______	Nasal:	______	______
Occipital:	______		Ethmoid:	______	
Temporal:	______	______	Lacrimal:	______	______
Zygomatic:	______	______	Vomer:	______	
Palatine:	______	______	Sphenoid:	______	

**Mandible**	Left	Right		Left	Right
Body:	______	______	Ramus:	______	______

**Postcranium**	Left	Right		Left	Right
Hyoid:	______		Thoracic 1-12 (count):	______	
Manubrium:	______		Lumbar 1-5 (count):	______	
Sternal Body:	______		Sacrum:	______	
Clavicle:	______	______	Ilium:	______	______
Scapula:	______	______	Pubis:	______	______
Humerus:	______	______	Ischium:	______	______
Radius:	______	______	Coccyx (count):	______	
Ulna:	______	______	Femur:	______	______
Ribs (count):	______	______	Patella:	______	______
Atlas:	______		Tibia:	______	______
Axis:	______		Fibula:	______	______
Cervical 3-7 (count):	______				

**Hands**	Left	Right		Left	Right
Scaphoid:	______	______	Capitate:	______	______
Lunate:	______	______	Hamate:	______	______
Triquetral:	______	______	Metacarpals (count):	______	______
Pisiform:	______	______	Prox phalanges (count):	______	
Trapezium:	______	______	Int phalanges (count):	______	
Trapezoid:	______	______	Dist phalanges (count):	______	

**Feet**	Left	Right		Left	Right
Calcaneus:	______	______	Medial cuneiform:	______	______
Talus:	______	______	Metatarsals (count):	______	______
Navicular:	______	______	Prox phalanges (count):	______	
Cuboid:	______	______	Int phalanges (count):	______	
Lateral Cuneiform:	______	______	Dist phalanges (count):	______	
Int. cuneiform:	______	______			

List the teeth that are present (Type, Upper vs Lower, Side):

Room for Notes if necessary:

Donation Number:__________
Analyst:__________
Date:__________

FACTS Forensic Team Biological Profile Requirement

This can be completed during coursework or outside of coursework. MA students need to complete Methods I and II with an 'A' before they can submit this form. PhD students can submit this form at anytime.

Step 1. Write up an Anthropology Report that includes a background, inventory, sex estimation, age estimation, biological affinity estimation, and stature estimation.

Step 2. Have a FACTS Team Leader approve your Anthropology Report by signing here:

I have reviewed the written anthropology report on case# ________________________ and approve the report for submission as the biological profile requirement for the FACTS Forensic Team.

____

Signature of Forensic Team Leader

Step 3. Submit the Paperwork from Step 1 and Step 2 for review as a single PDF document via email to facts@txstate.edu.

FACTS Forensic Team Co-Authored Report Requirement

PhD students can submit this form at anytime.

Step 1. Become a CAT Assistant Member.

Step 2. Co-Author two Anthropology reports with a FACTS Team Leader and have the Team Leader sign below.

Case No.	Printed name of Team Leader	Team Leader Signature

Step 3. Submit this form along with both reports as a single PDF document **on the** Canvas site under the CoAuthored Case Report assignment.
